# A Prospective Assessment of an Adjustable, Immediate Fit, Subischial Transfemoral Prosthesis

**DOI:** 10.1016/j.arrct.2022.100200

**Published:** 2022-05-02

**Authors:** Timothy R. Dillingham, Jessica L. Kenia, Frances S. Shofer, James S. Marschalek

**Affiliations:** a Department of Physical Medicine and Rehabilitation, University of Pennsylvania Perelman School of Medicine, Philadelphia, PA; b Department of Emergency Medicine, University of Pennsylvania Perelman School of Medicine, Philadelphia, PA; c Advanced Design Concepts, Pewaukee, WI

**Keywords:** Adjustable prosthesis, Amputation, Limb loss, Prosthetic, Transfemoral amputation, Transfemoral prosthesis, PCUQ, Prosthetic Comfort and Utility Questionnaire, PEQ, Prosthetic Evaluation Questionnaire

## Abstract

**Objective:**

To assess the feasibility of an adjustable, subischial transfemoral prosthesis by comparing self-reported outcome measures regarding socket comfort, fit and utility relative to a persons’ conventionally made socket. Assessing limb compressibility was another aim of this study.

**Design:**

A single-group pre-post intervention design.

**Setting:**

Physical medicine and rehabilitation biomechanics laboratory.

**Participants:**

All 18 enrolled participants (N = 18) completed the feasibility trial. There were 16 men and 2 women with an average age of 59.4 (±7) years. Most of the participants (61.1%) had worn a socket for 1 to 10 years before the trial, 22.2% of the participants had worn one for less than a year, and 16.7% of the participants had worn a prosthesis for more than 10 years.

**Intervention:**

Participants were fit with the study prosthesis and used it for a 2-week home trial.

**Main Outcome Measures:**

A Prosthetic Comfort and Utility Questionnaire was completed on the participant's conventional prosthetic device and the subischial socket system after the trial.

**Results:**

The adjustable subischial prostheses were rated superior overall to the participant's conventional sockets (40.9 ± 7.2 vs 32.8 ± 10.8; *P*=.004). Six of the 10 parameters measured (adjustability, overall fit, prosthesis weight, sitting comfort, standing comfort, and standing stability) were rated higher for the adjustable prostheses compared to the conventional sockets. Compression of the soft tissues of the thigh ranged from 5.6 ± 4.2 cm at the distal end to 7.3 ± 3.6 cm at the proximal site. There were no falls, skin breakdown, or limb ischemia. At the 2-month telephone follow-up, 61% of subjects had transitioned to using the adjustable subischial socket most of the time.

**Conclusions:**

The adjustable, immediate fit, subischial prosthesis provided safe, comfortable, and functional ambulation for persons with transfemoral limb loss in this short-term feasibility study. This study supports the consideration of a new paradigm in transfemoral prosthetics—adjustable subischial sockets. These devices should be tested in a larger multi-center study.

Traditional prosthesis fabrication often results in a hard socket that cannot easily be adjusted to accommodate residual limb changes. The typical transfemoral socket extends high up onto the leg— to the ischial tuberosity and ischial ramus area and are called quadrilateral or ischial containment sockets.^1^, ^2^, ^3^, ^4^, ^5^, ^6^, ^7^, ^8^ The residual limb changes significantly during the first year after amputation in size and shape.^9^ Limb volume fluctuates daily for many individuals, particularly those persons with renal and heart disease.^10^^,^^11^ Conventional hard sockets accommodate volume changes by adding or removing layers of socks to adjust socket volume—an often time-consuming process requiring the patient to find a private location and disrobe.

Many persons with lower limb loss report substantial discomfort and pain with their conventional sockets.^12^, ^13^, ^14^, ^15^ Data from a large, nationwide survey of prosthesis users (n=934, of whom 38.5% had transfemoral limb loss) who were members of the nonprofit advocacy group Amputee Coalition of America indicated that one-third of adult respondents reported discomfort with their prosthesis.^12^ Another study involving persons with traumatic amputation found that only 43% of the transfemoral participants reported satisfaction with prosthesis comfort.^13^ A survey by the U.S. Department of Veterans Affairs indicated that an average of 45% of people with transfemoral limb loss wished to change to a different socket type.^14^ Three out of 5 people reported seeking care from multiple prosthetists because they were dissatisfied with the fit of their conventional devices or the prosthetic services received.^15^

Comfort is, by far, the single most important characteristic correlated with successful prosthetic ambulation yet is often lacking.^12^, ^13^, ^14^, ^15^, ^16^, ^17^ Residual limb pain and skin issues are prominent issues that lead to reduced socket comfort among the adult population.^12^, ^13^, ^14^ Fully 63% of Operation Enduring Freedom/Operation Iraqi Freedom respondents reported skin problems on the residual limb and 54% of Vietnam War veteran respondents reported this problem as well.^14^ Prosthesis satisfaction and increased use also have been shown to significantly influence the likelihood of returning to work.^18^^,^^19^

The quadrilateral socket and the ischial containment (narrow mediolateral) sockets have been commonly used since about the 1970s.^1^, ^2^, ^3^ The posterior wall of the quadrilateral socket forms a flat area for weight bearing through the ischial tuberosity and gluteal muscles.^1^^,^^3^^,^^4^ The lateral wall is contoured over the greater trochanter and hip abductor muscle group.^5^ The ischial containment socket was developed to provide a more stable purchase on the limb.^5^, ^6^, ^7^ This design encloses the ischial tuberosity and ramus and places the femur in a more adducted position than the quadrilateral design.^6^^,^^7^ Both types of sockets have been found to restrict range of motion in the residual limb.^8^ A lower profile, subischial socket design has been described that uses hard socket technology and vacuum suspension.^20^, ^21^, ^22^, ^23^, ^24^

To enhance comfort and function an adjustable subischial transfemoral prosthesis was developed by iFIT Prosthetics, LLC ^a^ (fig 1). This socket was designed from experience gained through the development and commercialization of the transtibial immediate fit adjustable socket.^25^, ^26^, ^27^

**Fig 1:**
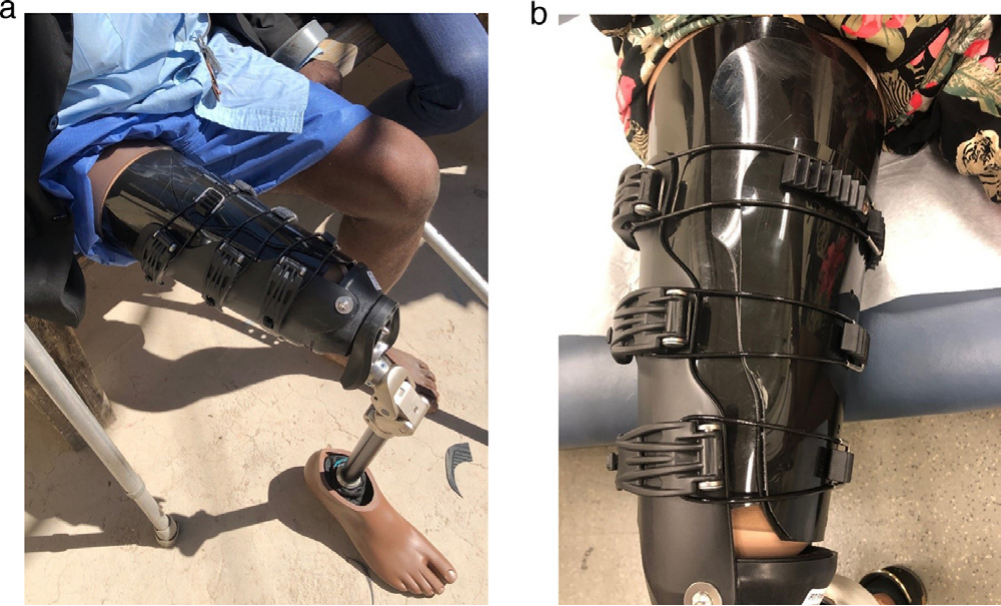
The locking buckle system for closure of the subischial socket. The buckles (situated on the lateral leg) and hooks (placed on the medial leg) close the adjustable socket securely around the limb.

The adjustable subischial transfemoral socket uses a buckle and cable system like the transtibial system to provide adjustability.^25^, ^26^, ^27^ The transfemoral system, however, features 3 buckles at the proximal, mid, and distal areas to accommodate volume changes and compressibility of the thigh soft tissues and will accommodate longer limbs. A silicone liner with locking pin attaches the socket to the patient's limb via shuttle lock.^28^ This is a common method for suspension where the pin engages securely with a lock in the base of the socket and is removed by pressing a release pin.

Like the transtibial adjustable socket, the transfemoral socket can be fitted and aligned using a few hand tools in a single setting. The socket features a low profile flexible inner liner with overlapping flaps that can be trimmed and heat molded to accommodate the patient (fig 2). The proximal trim line is subischial for enhanced sitting comfort and hip mobility. The subischial adjustable socket assessed in this study was designed for someone with recent amputation who will undergo changes in limb volume and for someone who wants adjustability throughout the day. It can be used as a preparatory prosthesis or a definitive device.

**Fig 2:**
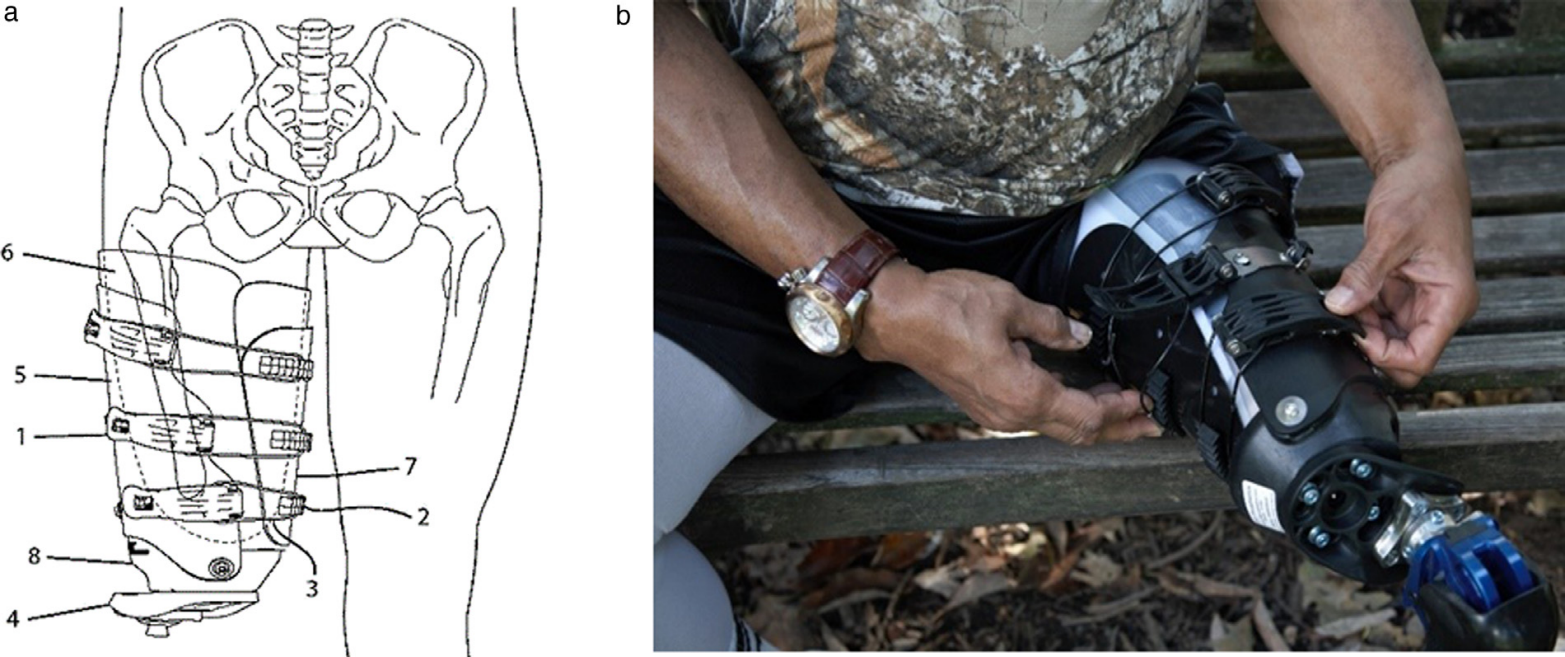
The adjustable subischial transfemoral prosthesis. Components: (1) locking buckle, (2) hook, (3) steel cable, (4) offset, (5) lateral rigid frame, (6) flexible/inner liner, (7) outer flap, and (8) cup.

The purpose of this study was to examine the feasibility of a subischial adjustable socket for people with transfemoral limb loss. The comfort, fit, and utility with these adjustable sockets were examined in a cohort of people with transfemoral limb loss. We hypothesized that the new adjustable sockets would provide safe and comfortable ambulation. Safety, comfort, and function were our primary outcomes. Another aim of this study was to assess the amount of compression in the socket once the participant adjusted the device to the preferred comfortable fit.

## Methods

People with transfemoral limb loss were recruited from the University of Pennsylvania health system through referrals, mailings, and advertisements in the local newspapers. Inclusion criteria consisted of unilateral transfemoral amputation, a well-healed limb, ability to ambulate using a prosthesis, weight under 260 pounds (estimated weight limit for socket componentry), and age 21 years and over. Exclusion criteria included open wounds or skin irritations, excessive limb pain, previous stroke, and brain injury that interfered with ambulation. This study was reviewed and approved by the University of Pennsylvania institutional review board, and all subjects gave written informed consent prior to participation. Subjects shown in this paper consented to have their images used in this manner.

All participants were consented by the research coordinator and fit by the principal investigator of the study in the Department of Physical Medicine and Rehabilitation Gait and Biomechanics Laboratory. The subjects had circumferential measurements taken of their limb at the distal third, midpoint, and proximal third, as well as from groin to end of residual limb. The measurements were taken on the skin directly and also while wearing a silicone pin suspension sleeve. Participants were asked to complete a Prosthetic Comfort and Utility Questionnaire (PCUQ) (see text box 1), which featured selected questions from the Prosthetic Evaluation Questionnaire (PEQ).^29^ The questionnaire was developed by selecting questions from the full PEQ to focus on aspects that pertained specifically to the socket such as comfort, fit, ambulation and utility. This strategy for assessing issues related to the prosthesis itself has been used by us in evaluation of transtibial prostheses.^25^^,^^26^ Another research team used a similar short questionnaire that was adapted from the full PEQ for studying 5 different prosthetic feet.^30^ We added specific questions regarding sitting comfort because this is an important aspect for persons with transfemoral limb loss. For ease of scoring, this questionnaire featured 10 questions each with a numeric rating scale from 1 (poor) to 5 (excellent), the same as was used by other investigators.^30^ The total points on each question were added to derive an overall patient satisfaction score (our primary outcome) with a possible range of scores from 10 to 50. Data were collected at the initial visit on the patient's conventional device and at the 2-week follow-up on the test prosthesis. Data were collected by the study coordinator without the principal investigator present in the room to minimize any influence on the patient's perceptions of the devices.

Two adjustable socket sizes—standard and wide—were developed to encompass most people with transfemoral limb loss. The subjects were all given a silicone locking sleeve to wear. The prosthesis was suspended by a pin suspension system using the silicone sleeve. Subjects were placed on an exam table while the socket was placed on the residual limb to estimate how much to trim the proximal brim (inner liner). Excess material was trimmed so that the socket fit such that the proximal brim was below the ischial tuberosity and ramus. After trimming and smoothing the edges of the socket, it was placed back on the residual limb and tightened using the buckle system until a snug yet comfortable fit was achieved. The trim line was again re-evaluated while standing to insure that it was fully subischial. One of 2 different knees were used. An OFM2 Balance Knee from Ossur^b^ was used for persons with K1 and K2 ambulation levels,^31^ while the Ossur Mauch knee was used for persons that were at K3 or K4 ambulation levels. This was determined by the patient's description of their activity levels, as well as by assessing the subject's current knee and whether they required assistive gait devices such as a walker or cane. The participant's own prosthetic knee was left on their conventional device as a safety precaution. In case any issue was encountered with the adjustable subischial test device, they would always have their conventional prosthesis available and in good working order for safe ambulation. An Alps^c^ 3mm AKHD silicone locking liner and a College Park^d^ Breeze foot were used for all subjects. Two rectangles measuring 6 by 4 inches of anti-rotation material were placed within the socket on the inside of the flexible liner to prevent rotation between the residual limb and the device when ambulating.

At the end of the initial fitting, circumferences of internal socket diameter were also measured. Measurements of the internal socket were taken using an Ottobock^d^ Inside Circumference Gauge (Salt Lake City, UT). The internal diameter of the socket was marked in the position (buckle closure) that the patient found most comfortable and functional. After removing the device, the circumferences of the inner socket were measured at the distal third, midpoint, and proximal third of the inner socket with the socket closed at the position of the marks corresponding to a comfortable secure fit. These were then compared to the residual limb circumferences with and without the silicone suspension sleeve on the limb.

Commercially available offset adaptors were initially used to achieve an optimal knee alignment. Later in the trial, an injection molded offset was designed and manufactured by our team that matched the socket design to provide the necessary socket flexion and knee offset for stable gait. The offset is attached to the socket cup. This offset provides rotation at both the bottom of the cup and through a rotatable pyramid adapter attached to the bottom for accepting the knee unit. The prosthesis was assembled, fit, and aligned to provide a stable base of support for comfortable and safe ambulation with the hip in approximately 5° of flexion. Once subjects demonstrated they were able to properly put on and take off the device, as well as walk proficiently, they were cleared to take the prosthesis home for a 2-week trial.

At the initial visit, the subjects rated their conventional prostheses. At the 2-week follow up appointment after the subjects used the adjustable subischial transfemoral prosthesis, they completed the PCUQ. This information was collected by the study coordinator without the principal investigator present. Each participant had their residual limb inspected for skin irritation or wounds. Any report of a fall by the subject or breakage of the prosthetic socket or prosthetic component was recorded. They were allowed to keep the adjustable prosthesis (socket, knee unit, pylon, and foot) if they chose to do so after the study. Participants who kept the prosthesis were followed up through telephone call after 2 months to determine whether they were still wearing the adjustable prosthesis.

**Fig 3:**
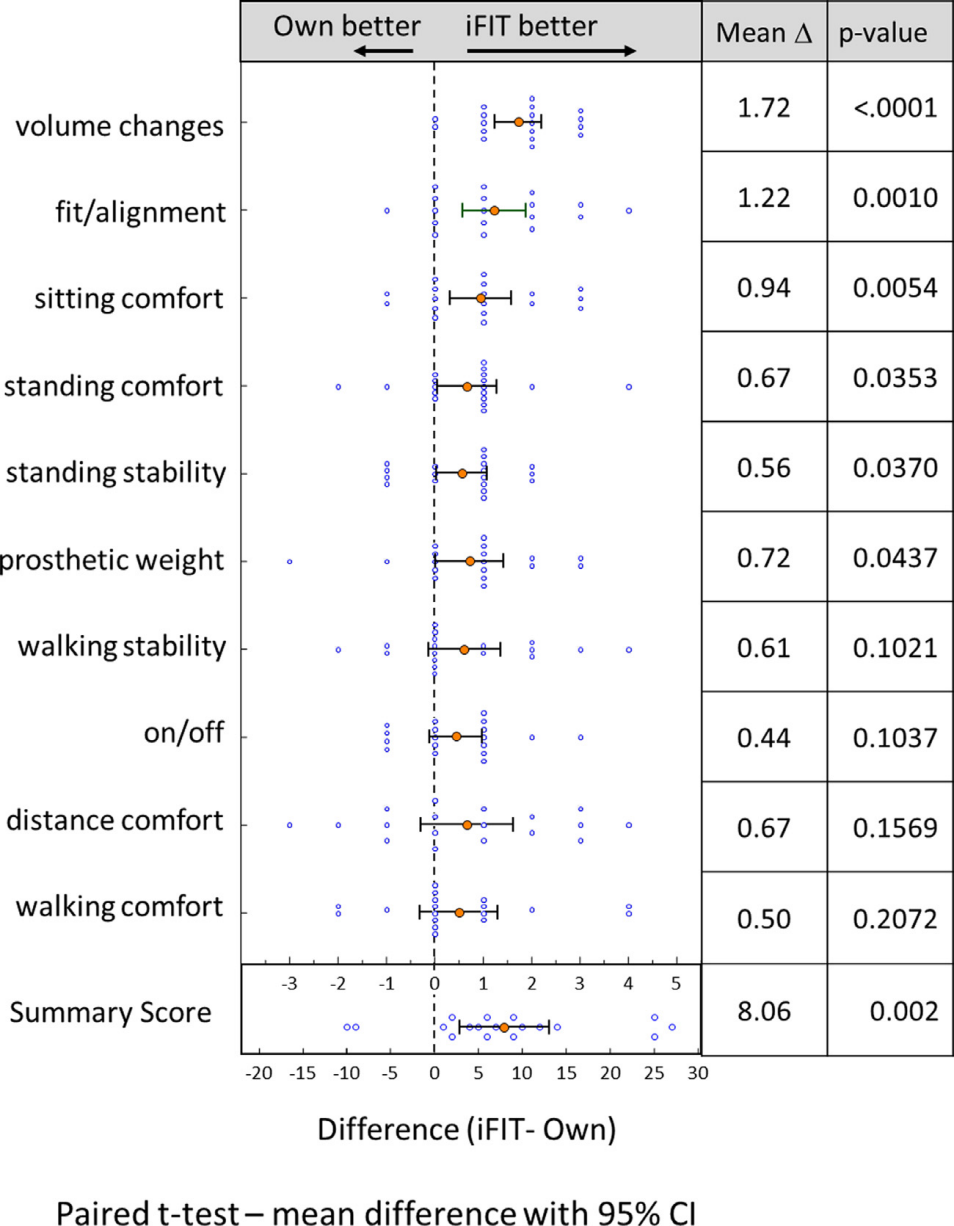
Results of the Socket Comfort and Utility Questionnaire comparing conventional socket (own) and immediate fit socket (iFIT) after 2-week home trial. CI, confidence interval.

A sample size of 12 to 14 was predicted to achieve >80% power, with an alpha of 0.05. using a clinically meaningful difference of 1 (effect size) on an individual PCUQ question (range of score, 1-5) with SD of the mean difference as large as 1.3. To describe the study population, frequencies and percentages were calculated for categorical variables, and means and standard deviations were calculated for continuous variables. To compare responses to the individual PCUQ questions and the patient satisfaction score between the iFIT device and patients’ conventional device, paired t-tests were used because the mean differences were approximately normally distributed, with only mild left skew and lightly tailed. Results are presented as mean difference ± 95% confidence intervals between the adjustable socket and the conventional device. All analyses were performed using SAS statistical software (version 9.4, SAS Institute, Cary, NC). Data were collected by the study coordinator and maintained by her independent of the principal investigator. All statistical analyzes were performed by the biostatistician (F.S.) independent of the principal investigator. Figure 3 was created using GraphPad Prism (version 9.2, GraphPad Software, Inc, San Diego, CA).

## Results

Eighteen people with transfemoral limb loss volunteered to participate in the study, and none dropped out of the study or were lost to follow-up. There were 16 men and 2 women who participated and completed the study with a mean age of 59.4 (±7.0) years (table 1). Fifty-six percent were white, 33% were African American, and 11% were Hispanic. Most of the participants (5%) wore a suction socket system with silicone suction liner and had limb loss resulting from dysvascular disease (55.6%).

**Table 1:** Description of Participants: Variables for 18 Enrolled Participants

Demographics		N	Percent
Sex	Men	16	89
	Women	2	11.1
Race/ethnicity	White	10	55.6
	African American	6	33.3
	Hispanic	2	11.1
Etiology of limb loss	Dysvascular	10	55.6
	Traumatic	5	27.8
	Cancer	3	16.7
Type of conventional socket suspension (all sockets were non-adjustable hard sockets)	Suction (silicone sleeve)	9	50
	Suction (Skin suction)	1	5.6
	Lanyard	6	33.3
	Pin	1	5.6
	Strap/waist belt	1	5.6
Type of prosthetic knee on conventional socket	Mechanical knee	7	39
	Computerized knee	11	61
Length of time wearing a prosthesis	Less than 1 year	4	22.2
	1-10 years	11	61.1
	>10 years	3	16.7
Average time wearing the conventional prosthesis during a typical day	9 or more hours	11	61.1
	7-9 hours	0	0
	4-6 hours	2	11.1
	1 to 3 hours	5	27.8
Average time wearing the adjustable prosthesis during a typical day	9 or more hours	9	50
	7-9 hours	1	5.5
	4-6 hours	3	16.7
	1-3 hours	5	27.8

Participants rated the adjustable subischial transfemoral prosthesis significantly better than the conventional prosthesis on the summary satisfaction score PCUQ survey (40.9 vs 32.9; *P*=.004). For all 10 questions, the adjustable prosthesis device was rated better than the conventional prosthesis (mean difference for all 10 questions=0.81±0.39) and for 6 of the questions (volume changes, adjustability, prosthesis weight, sitting comfort, standing comfort, and standing stability), the adjustable subischial prosthesis was rated significantly better (*P*<.05) (fig 3). The overall score was rated better for the adjustable socket than for the conventional prosthesis in 16 of the 18 participants. Daily wear time was not significantly different between the sockets despite differences in knee units between the test devices and what the subjects normally wore.

The thigh tissue compressibility showed large differences between the residual limb measurements and the adjustable socket internal circumferences (table 2). These differences were 7.3 cm ±3.6 proximally, 7.2 cm ±3.9 at midpoint, and 5.6 cm ±4.2 at the distal end while wearing a liner (see table 2).

**Table 2:** Comparison of Residual Limb Circumference Measurements to Internal Socket Diameter to Assess Compressibility of Residual Limb Tissue

Type of Residual Limb	Average Circumference Without Suspension Sleeve (cm)	Average Circumference Wearing Suspension Sleeve (cm)	Average Internal Socket Circumference (cm)	Average Difference Between Limb (with Suspension Sleeve) and Socket Circumference (cm)
Proximal third residual limb	51.7 (7.2)	53.6 (SD 7.2)	46.3 (SD 5.3)	7.3 (SD 3.6)
Midpoint residual limb	47.2 (7.7)	47.7 (SD 6.1)	40.5 (SD 4.9)	7.2 (SD 3.9)
Distal third residual limb	40.6 (7.0)	41.3 (SD 5.9)	35.7 (SD 5.6)	5.6 (SD 4.2)

NOTE. A cohort of 18 people with unilateral transfemoral limb loss tested an immediate fit, adjustable subischial prosthesis for a 2-week trial. The test prosthesis was rated as superior to their conventional prostheses in adjustability, overall fit, prosthesis weight, sitting comfort, standing comfort and standing stability, as well as the overall satisfaction score. In this cohort, 61% of the subjects transitioned to using the adjustable socket full time. Thigh tissue demonstrated a high level of compressibility. This new subischial socket is feasible and safe for use in persons with transfemoral limb loss.

**Fig 4:**
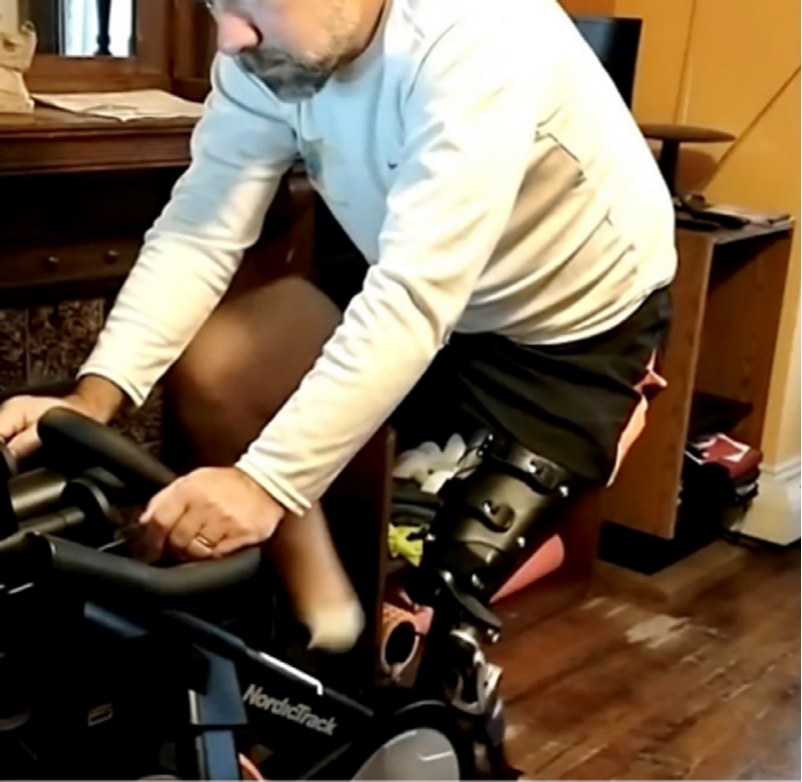
The subischial socket with its low profile on the leg, allowed this subject to comfortably ride a stationary bike.

None of the participants reported a fall, skin breakdown, or other adverse event. There were no component failures with the adjustable devices. One subject started biking for exercise again because his sitting comfort was improved (fig 4).

All but 1 participant who completed the home trial elected to keep the adjustable prosthesis. The participant who did not keep the prosthesis was getting a revision surgery. At the 2-month follow up, 11 of the 18 participants (61%) reported that they switched to wearing the adjustable socket for most of the time. The other 6 participants reported that they wore the adjustable socket interchangeably with their conventional device.

## Discussion

In this feasibility study, the adjustable, subischial transfemoral prosthesis was shown to be safe, comfortable, and feasible as a potential alternative to conventional sockets. Subjects rated the adjustable subischial socket as significantly better than conventional prostheses on the primary outcome—the overall PCUQ survey score. The results of this study are similar to those found when comparing adjustable transtibial prostheses to conventional transtibial prostheses.^25^^,^^26^ The adjustable subischial socket appeared to accommodate thigh tissue compression that occurs within the socket.

For 6 of the 10 questions (see fig 3), the adjustable prosthesis was rated as significantly better. The ability to accommodate volume changes had the highest difference in favor of the adjustable subischial socket, which is not surprising, because most of the participants used a rigid conventional socket, which has limited ability to accommodate volume changes. All participants were able to use the pin suspension system and locking buckle mechanisms to fit the socket in a secure and comfortable manner. Sitting comfort was enhanced, with significantly better scores noted for the subischial sockets. This can be attributed to the lower profile brim that did not extend up into the pelvic region (ischial ramus or ischial tuberosity).

Most of the conventional sockets (55.6%) worn by the participants had suction suspension systems, followed by lanyard suspension (33.3%). Subjects found no significant differences in taking the prosthesis off and putting it on with the pin suspension system used in this trial.

At the end of the study, participants were asked if they wanted to keep their adjustable prostheses. All of the subjects, except for 1 who reported getting limb revision surgery, kept their prosthesis. In our follow-up of this cohort after 2 months, 61% had switched to using the adjustable subischial prosthesis for most of the time. Some had their computerized knees put onto the adjustable socket. These results suggest that this subischial adjustable prosthesis may be a feasible option for many transfemoral prosthetic users.

The adjustable subischial transfemoral prosthesis was rated as more comfortable (sitting and standing), according to self-reported outcome scores. Comfort, an important factor in prosthesis satisfaction and use, is an important consideration when choosing the right system for each individual.^12^, ^13^, ^14^, ^15^, ^16^ The average person with limb loss visits the prosthetist 9 times per year and requires a new socket every 1 to 2 years.^12^^,^^13^ With an adjustable socket, these visits can likely be reduced, because the patient can themselves make size adjustments to accommodate changes in limb size and shape. Considerable changes in volume occur after the first year after amputation. Socket adjustability can help accommodate these changes more readily. Persons with heart and renal failure also tend to exhibit daily volume fluctuations and could find an adjustable socket useful for maintaining a better fit throughout the day.

The componentry in the adjustable sockets provides strength and compliance for a wide range of limb circumferences and lengths. They are mass produced using injection molding technology, which provides consistent quality and strength.

With the adjustable socket, prosthesis fitting and gait training can begin when surgical wounds are healed. Even with edema and bulbous residual limbs, patients can begin rehabilitation and simply adjust the socket as the limb matures and edema resolves. This prosthesis works with most commercially available feet and knee units.

Early ambulation can be advantageous as it helps to minimize joint contractures and deconditioning, and is associated with higher levels of prosthesis use.^32^^,^^33^ A recent study by Miller et al found that receiving a prosthesis earlier, within the first 3 months’ post-amputation, reduced health care costs by $25,000 during the following year.^32^ Past studies have found that only 26%^34^ to 57%^35^ of people with a transfemoral amputation receive a prosthesis. A study in the U.S. Department of Veterans Affairs found that patients who received a prosthesis within a year from amputation had a 25% 3-year mortality rate versus 45% for those who did not receive a prosthesis.^36^ Decreased wait time for getting a comfortable and functional prosthesis is linked to increased satisfaction, higher usage, and overall better long term outcomes.^12^^,^^15^^,^^37^

Another group that may benefit from an adjustable socket are children with limb deficiencies who report frequent problems with conventional sockets as they grow.^38^, ^39^, ^40^ Boonstra et al found that fully 74% of children and their parents reported skin issues within the prosthesis as a problem and that 22% had skin breakdown at the time of the study.^40^ The reason for these problems was described by these researchers as “ill-fitting prostheses.”^40^ There is a high care burden for families caring for pediatric patients with lower limb loss. Total travel time, prosthetist visit time, and therapy time for pediatric patients and their families averaged 42.7 hours a year, according to one study.^38^ Adjustable sockets that accommodate growth could address these problems.

The adjustable subischial prosthesis described in this feasibility study represents a departure from conventional socket shapes and biomechanical principles. In contrast to quadrilateral and ischial containment sockets, which extend far up the residual limb into the groin and perineum, the adjustable sockets are lower in profile, extending up the leg to a point well below the ischium. The soft tissues are firmly yet comfortably grasped with the adjustable socket, and, according to the subject's self-reported outcome measures, provide a stable base of support for safe stable ambulation.

Even though the sockets are flexible and adjustable, they provide sufficient strength and durability for the user. These injection-molded sockets underwent cyclic testing using the International Standardization Organization specifications. They exceeded these standards for strength and durability.^41^ Experience in Jamaica demonstrated that similar adjustable prostheses (transtibial) are durable even in demanding environments with daily use.^27^

The level of thigh soft tissue compressibility discovered in this study suggests that conventional sockets using limb casts or digital limb scans with 3-dimensional printing to create the hard socket, are unlikely to accurately predict the proper accommodation of soft tissue limb compression. Previous researchers designed subischial sockets made from limb casts that were made smaller than the limb.^20^, ^21^, ^22^, ^23^, ^24^ They then used suction suspension to pull the soft tissues into the socket. These investigators demonstrated modest utility with this approach.^22^, ^23^, ^24^

Many conventional transfemoral prostheses are unaffordable to the large segment of the population who are under- or uninsured and lack the financial resources to cover the devices’ out-of-pocket costs. A study by Mackenzie et al found the average cost of an above the knee prosthesis is $18,744, with patients needing a new prosthesis every 2.3 years.^38^ Insurance companies are often reluctant to reimburse for multiple socket revisions for a person with a changing residual limb. The adjustable subischial transfemoral socket is less expensive than conventional sockets, providing a more accessible option for many patients as either a preparatory device to accommodate loss of limb volume after surgery or as a definitive device.

There were notable strengths of this feasibility study. The outcome measures assessed important aspects of socket comfort and utility. Significant findings across multiple questions and the overall significant score with a sample of 18 subjects is compelling. We had a racially diverse sample, although most were men. Subjects rated the adjustable subischial transfemoral socket as better than conventional sockets in our main outcome measure and subcategories of comfort and function. These represent meaningful aspects of prosthesis use and patient satisfaction. None of the subjects had difficulty buckling or removing the socket. At the 2-month follow-up, 61% of subjects altered their prosthesis use in favor of wearing the adjustable socket the majority of time. This is further endorsement of the usefulness and acceptance of this new socket technology.

### Study limitations

There are several limitations in the present study. This is a study from a single institution. Given the nature of the study, subjects were not blinded as to the prosthesis they were using. The study used a sample of convenience, and participants who entered the study might have been less satisfied with their devices than a general population of prosthesis users. Subjects were able to keep the prosthesis after the study, and this might have introduced some bias. We were not able to completely match the knee units for participants who wore computerized knees on their conventional devices. However, this did not seem to affect the scoring of the socket system, the daily ambulation with the devices, or the patients’ willingness to continue using these study prostheses after the trial. Our subjects used the knees we provided them in a safe manner and demonstrated safe and functional ambulation. The questionnaire used in the study was adapted from the Prosthetic Evaluation Questionnaire, and therefore has not been fully validated. We addressed to the extent possible, issues of potential influence by the principal investigator. The principal investigator was not present when collecting data. All statistical analyses were conducted by an independent statistician (F.S.) on the team who resides in a separate department.

## Conclusions

The adjustable subischial transfemoral prostheses in this study demonstrated feasibility and safety for use by persons with transfemoral limb loss. Residual limbs demonstrated a high degree of tissue compressibility. This study provides evidence to support the consideration of these devices for persons with transfemoral limb loss. They should be evaluated in a larger multi-center study.

**Table:** 

Text Box 1. Prosthetic Comfort and Utility Questionnaire
Characteristics of the prototype prosthesis. Rated on a scale of 1 through 5: 1, Poor; 2, Below Average; 3, Average; 4, Above Average; 5, Excellent.
1. Comfort while standing
2. Comfort while walking short distances
3. Comfort while walking long distances
4. Comfort while sitting
5. Weight of the prosthesis
6. Stability while standing
7. Stability while walking
8. Taking the prosthesis off and putting it on
9. The ability of your device to accommodate volume changes
10. How satisfied are you with the overall fit and alignment of this prosthesis?

## Suppliers

a. iFIT Prosthetics LLC- iFIT TF Prosthesis. b. Ossur Americas- OFM2 Knee, Mauch. c. Alps- AKHD gel locking liner. d. College Park Industries- Breeze foot. e. Ottobock- Inside circumference gauge.

## Appendix Supplementary materials

Image, application 1
